# Ferroelectric Field Effect Induced Asymmetric Resistive Switching Effect in BaTiO_3_/Nb:SrTiO_3_ Epitaxial Heterojunctions

**DOI:** 10.1186/s11671-018-2513-6

**Published:** 2018-04-13

**Authors:** Caihong Jia, Jiachen Li, Guang Yang, Yonghai Chen, Weifeng Zhang

**Affiliations:** 1 Henan Key Laboratory of Photovoltaic Materials, Laboratory of Low-Dimensional Materials Science, School of Physics and Electronics, Henan University, Kaifeng, 475004 People’s Republic of China; 2 Key Laboratory of Semiconductor Materials, Institute of Semiconductors, Chinese Academy of Sciences, Beijing, 100083 People’s Republic of China; 3 College of Materials Science and Opto-Electronic Technology, University of Chinese Academy of Sciences, Beijing, 100049 People’s Republic of China

**Keywords:** Ferroelectric, Asymmetric resistive switching, Ferroelectric/semiconductor heterojunctions

## Abstract

Asymmetric resistive switching processes were observed in BaTiO_3_/Nb:SrTiO_3_ epitaxial heterojunctions. The SET switching time from the high-resistance state to low-resistance state is in the range of 10 ns under + 8 V bias, while the RESET switching time from the low-resistance state to high-resistance state is in the range of 10^5^ ns under − 8 V bias. The ferroelectric polarization screening controlled by electrons and oxygen vacancies at the BaTiO_3_/Nb:SrTiO_3_ heterointerface is proposed to understand this switching time difference. This switch with fast SET and slow RESET transition may have potential applications in some special regions.

## Background

Ferroelectric resistive switching effects have attracted lots of research interests, since the polarization reversal is based on purely electronic mechanism, which does not induce a chemical alteration and is an intrinsically fast phenomenon [1, 2]. Ferroelectric resistive switching effects have been observed in ferroelectric heterojunctions sandwiched by two metal or semiconductor electrodes [3–5]. Lots of interesting behaviors have been observed in ferroelectric/semiconductor heterojunctions. For example, a greatly enhanced tunneling electroresistance is observed in BaTiO_3_ (BTO)/(001)Nb:SrTiO_3_ (NSTO) [4, 5] and MoS_2_/BaTiO_3_/SrRuO_3_ [6] heterojunctions since both the barrier height and width can be modulated by ferroelectric field effect. A coexistence of the bipolar resistive switching and negative differential resistance has been found in BaTiO_3_/(111)Nb:SrTiO_3_ heterojunctions [7]. The optically controlled electroresistance and electrically controlled photovoltage were observed in Sm_0.1_Bi_0.9_FeO_3_/(001)Nb:SrTiO_3_ heterojunctions [8]. A ferroelectric polarization-modulated band bending was observed in the BiFeO_3_/(100)NbSrTiO_3_ heterointerface by scanning tunneling microscopy and spectroscopy [9]. A transition from the rectification effect to the bipolar resistive switching effect was observed in BaTiO_3_/ZnO heterojunctions [10].

Here we observe an asymmetric resistive switching effect in the BaTiO_3_/Nb:SrTiO_3_ Schottky junction, which has not been reported yet. Furthermore, we propose a ferroelectric field effect to understand this asymmetric resistive switching effect. Specifically, the SET transition from the high- to low-resistance state is in 10 ns under + 8 V bias, while the RESET transition from the low- to high-resistance state is in the range of 10^5^ ns under − 8 V. This can be understood by the ferroelectric polarization screening by electrons and oxygen vacancies at the BaTiO_3_/Nb:SrTiO_3_ interface. This switch with fast SET and slow RESET transitions may have potential applications in some special regions.

## Methods

The commercial (100) 0.7 wt% NSTO substrates were successively cleaned in 15 min with ethanol, acetone, and de-ionized water and then blown with air before deposition. The BTO film was grown on NSTO substrates by pulsed laser deposition (PLD) using a KrF excimer laser (248 nm, 25 ns pulse duration, COMPexPro201, Coherent) at an energy of 300 mJ and frequency of 5 Hz, with the base vacuum of 2 × 10^−4^ Pa. During growth, the substrate temperature was kept at 700 °C, and the target-substrate distance was 6.5 cm. The oxygen partial pressure was 1 Pa, and the growth time was 15 min. After growth, the sample was kept under the oxygen partial pressure of 1 Pa for 10 min, and then, the temperature was reduced to room temperature at 10 °C/min within a vacuum environment. The thickness of BTO thin films is around 100 nm. Au top electrodes (0.04 mm^2^) were sputtered on BTO thin films through a shadow mask by DC magnetron sputtering, and the bottom electrode was indium (In) pressed on NSTO substrate. Keithley 2400 sourcemeter was used to conduct transport measurements. Voltage pulses were supplied by an arbitrary waveform generator (Agilent 33250A) with a pulse duration ranging from 10 ns to 1 s. The atomic force microscopy (AFM), piezoresponse force microscopy (PFM), and scanning Kelvin probe microscopy (SKPM) results were carried out to characterize the morphology, ferroelectricity, and electrostatic potential of the BTO film surface by an Oxford AR instrument. The PFM out-of-plane phase, PFM out-of-plane amplitude, current, and SKPM images were recorded with a biased conductive tip of 0.5 V over the same area after writing an area of 2 × 2 μm^2^ with − 8 V and then the central 1.25 × 1.25 μm^2^ square with + 8 V. In all measurements, the bottom electrodes were grounded and voltages were applied onto the top electrodes or the tip. All measurements were performed at room temperature.

## Results and Discussion

**Fig. 1:**
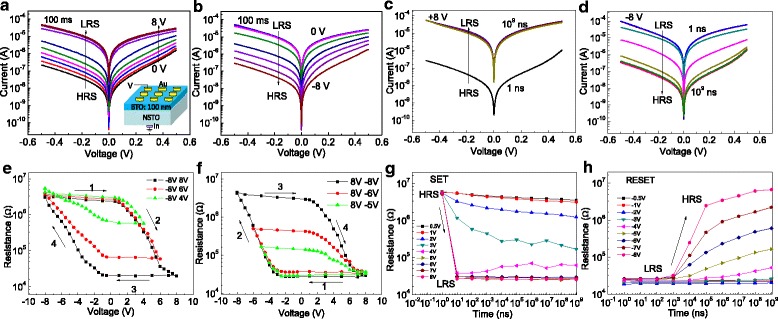
The current-voltage curves of the Au/BTO/NSTO/In system at small biases between − 0.5 and 0.5 V after applying a pulse in width of 100 ms with various amplitudes (**a**, **b**). The current-voltage curves of the Au/BTO/NSTO/In system at small biases after applying a pulse with an amplitude of + 8 V (**c**) and − 8 V (**d**) with various pulse widths. The junction resistance of the Au/BTO/NSTO/In system recorded at − 0.3 V after the application of voltage pulses with various amplitudes and widths starting from the HRS (**e**, **g**) or LRS (**f**, **h**), where the pulse widths in **e** and **f** are 100 ms and the different curves in **e** and **f** correspond to different consecutive measurements, with varying positive or negative maximum voltages. The inset of **a** shows the schematic drawing of the device structure

Figure 1a–d shows the current-voltage curves of the Au/BTO/NSTO/In system measured at small biases between − 0.5 and 0.5 V after applying a pulse with different amplitudes and widths, in which Fig. 1a, b is measured after pulses in width of 100 ms with various amplitudes, while Fig. 1c, d is measured after pulses in amplitudes of + 8 and − 8 V with various widths, respectively. Figure 1e–h shows the junction resistance recorded at − 0.3 V after the application of voltage pulses with different amplitudes and widths starting from the high-resistance state (HRS) (Fig. 1e, g) or low-resistance state (LRS) (Fig. 1f, h), where the pulse widths in panels e and f are 100 ms and the different curves in panels e and f correspond to different consecutive measurements, with varying positive or negative maximum voltages. The inset of Fig. 1a shows the schematic drawing of the device structure. The resistive switching in Au/BTO/NSTO is demonstrated by the current-voltage curves at small bias and the resistance loops as a function of the writing pulse amplitudes, after a relatively long 100-ms pulse was first applied with varying amplitudes from − 8 to 8 V, as shown in Fig. 1a, b, e, f. Obviously, the positive pulses can set the device to the low-resistance state, whereas the negative pulses switch the device back to the high-resistance state. Interestingly, both the switchings between the ON and OFF states are gradual, which is helpful for multistate resistance switching devices no matter it starts from HRS or LRS. These gradual transitions between the HRS and LRS were also observed in the BTO/La_0.67_Sr_0.33_MnO_3_ ferroelectric tunnel junction [2]. A hysteresis cycle between the low- (3 × 10^4^ Ω) and high- (3 × 10^6^ Ω) resistance states is observed, with a large OFF/ON ratio of 100 when the write voltage is swept between + 8 and − 8 V (Fig. 1e, f, black curves). The minor loops in Fig. 1e, f show that the final resistance state can be finely tuned between the HRS and LRS depending on the cycling protocol. Similarly, a writing pulse with varying amplitude from − 8 to 8 V and width from 10 ns to 1 s was applied to the device, and the I-V curves and junction resistance were subsequently recorded as a function of the writing pulse width, as shown in Fig. 1c, d, g, h. Obviously, switching between the HRS and LRS occurs only when the positive (negative) voltage pulse duration is long enough and the amplitude is large enough. For both the SET and RESET processes, the pulse duration is getting smaller with increasing the absolute pulse voltage amplitude. Specifically, the switching time from the HRS to LRS is remarkably fast, in which 10-ns pulses above 4 V are sufficient to saturate the junction resistance, as shown in Fig. 1g. In contrast, full switching to the HRS is only accomplished by relatively long RESET pulses on the timescale of milliseconds, as shown in Fig. 1h. For the application of memristive devices, Fig. 1e–h also shows that multilevel operation can be achieved by programming pulse voltage amplitude or duration.

**Fig. 2:**
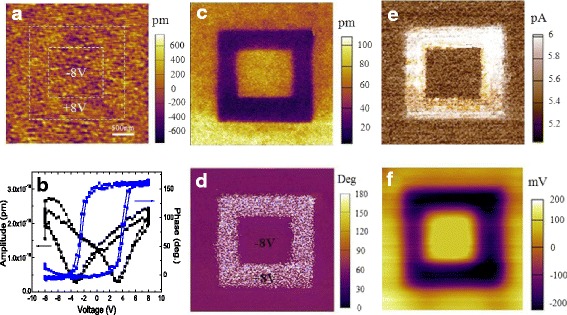
**a** Surface morphology of the BTO films on NSTO substrates. **b** Local PFM out-of-plane hysteresis loops: blue, phase signal; black, amplitude signal. **c** PFM out-of-plane phase, **d** PFM out-of-plane amplitude, **e** current, and **f** SKPM images recorded over the same area (**a**) after writing an area of 2 × 2 μm^2^ with − 8 V and then the central 1.25 × 1.25 μm^2^ square with + 8 V using a biased conductive tip. The scale bar is 500 nm for the images of **a** and **c**–**f**. The labels in **c**–**e** are corresponding to the value of out-of-plane current, PFM phase, and PFM amplitude, respectively

Topography images in Fig. 2a show that the BTO film surface is atomically flat, which prevents short circuits between the top and bottom electrodes [11]. Piezoresponse force microscopy (PFM) out-of-plane hysteresis loops shown in Fig. 2b indicate the ferroelectric nature of the BTO films. The local coercive voltages are about + 3.1 and − 3.1 V, indicated by the minima of the amplitude loop, as shown in Fig. 2b. Figure 2c–f shows the PFM out-of-plane phase, PFM out-of-plane amplitude, current, and SKPM images of ferroelectric domains written on the BTO surface recorded over the same area in Fig. 2a after writing an area of 2 × 2 μm^2^ with + 8 V and then the central 1.25 × 1.25 μm^2^ square with − 8 V using a biased conductive tip. A smaller (larger) current is observed over the central (outer) domain with a ferroelectric polarization pointing away from (to) the semiconductor substrate when the BTO is poled by − 8 V (+ 8 V). This has been used as an essential evidence to demonstrate the polarization-dependent resistive switching effect in ferroelectric heterojunctions [4]. Furthermore, it can be seen that the conduction in both the HRS and LRS are quite uniform, so there are no conductive filaments formed. According to the principle of SKPM, it measures two-dimensional distributions of contact potential difference between the tip and the sample with resolution in the nanometer range. The contact potential difference can be converted to the work function of the sample if the measurement is performed under thermoequilibrium state, and it is the electrical potential when a bias is applied to the sample. Thus, a positive (negative) tip bias would attract the negative (positive) ions and/or polarization charges to the surface, making the surface potential lower (higher) [12]. This prediction is consistent with our observations in Fig. 2f, confirming the variations of polarization charges as the major effects. Thus, the resistance switching in BTO/NSTO heterojunctions can be understood by ferroelectric polarization reversal, which has also been discussed in our previous reports [13]. However, the operation speed for both SET and RESET should be in the same order of 10 ns for purely ferroelectric polarization reversal [2], which is opposite to our observations of four-order difference between SET and RESET speed, as shown in Fig. 1g, h. Then a question comes with how to understand the operation speed difference of SET and RESET?

The apparent asymmetry in switching time has also been observed in Al/W:AlO_x_/WO_y_/W [14], La_2/3_Sr_1/3_MnO_3_/Pb(Zr_0.2_Ti_0.8_)O_3_/La_2/3_Sr_1/3_MnO_3_ [15], and Pt/LaAlO_3_/SrTiO_3_ [16] devices. Wu et al. proposed an asymmetric redox reaction in W:AlO_x_/WO_y_ bilayer devices and attributed the switching time difference to the different Gibbs free energy in AlO_x_ and WO_y_ layers [14]. However, in the present BTO/NSTO heterojunction, the voltage can only be applied to BTO film since NSTO is a heavily doped semiconductor. Thus, the asymmetric redox reaction can be ruled out in the present work. Qin et al. and Wu et al. attribute the asymmetry in switching time to the different internal electric field that drives oxygen vacancy migration across the LSMO/Pb(Zr_0.2_Ti_0.8_)O_3_ and LaAlO_3_/SrTiO_3_ interfaces [15, 16]. According to this model of oxygen vacancies across interface, the oxygen vacancy will migrate from BTO to NSTO under a positive bias, and the resistance in BTO will increase due to the decrease of oxygen vacancy concentration in BTO, while the resistance in NSTO will not change much since it already has high concentration of Nb donors; thus, the resistance of the whole system will increase under positive bias, which is opposite to our observation in Fig. 1. Furthermore, the ionic process is supposed to be much slower than the electron process, so a pure ion process cannot account for the fast SET process of 10 ns, as shown in Fig. 2g. Therefore, it is hard to understand the asymmetric resistive switching speed by only considering the physical process of polarization reversal or chemical process of drifted oxygen vacancies. Actually, an asymmetric switching speed has also been observed in Au/NSTO [17] and ZnO/NSTO Schottky junctions [18]. An asymmetric Schottky barrier can also lead to an asymmetric resistive switching speed. However, based on the PFM and SKPM results, the resistive switching in the BTO/NSTO heterojunction in the present work is observed to be caused by ferroelectric field effect. Therefore, we propose a model of ferroelectric polarization reversal coupled with the migration of oxygen vacancy across the BTO/NSTO interface to understand this asymmetric behavior.

**Fig. 3:**
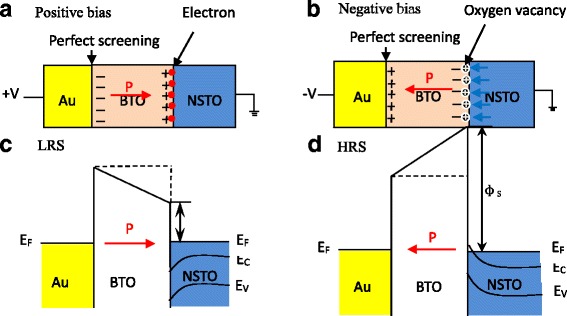
The schematic drawings (**a**, **b**) and corresponding potential energy profiles (**c**, **d**) of the Au/BTO/NSTO structures for the low- and high-resistance states. In BTO, the red arrows denote the polarization directions, and the “plus” and “minus” symbols represent positive and negative ferroelectric bound charges, respectively. The “circled plus” symbols represent the ionized oxygen vacancies. The blue arrows show the direction of oxygen vacancies drifting across the BTO/NSTO interface

Figure 3 shows schematic drawings (Fig. 3a, b) and corresponding potential energy profiles (Fig. 3c, d) of the Au/BTO/NSTO structures for the low- and high-resistance states. In BTO, the red arrows denote the polarization directions and the “plus” and “minus” symbols represent positive and negative ferroelectric bound charges, respectively. The “circled plus” symbols represent the ionized oxygen vacancies. The blue arrows show the direction of oxygen vacancies drifting across the BTO/NSTO interface. For simplification, we assume that the ferroelectric bound charges at the Au/BTO interface can be perfectly screened. Therefore, the barrier height at the Au/BTO interface is fixed and does not change with the polarization reversal. The barrier height at the BTO/NSTO interface will become smaller (larger) with polarization pointing to the bottom (top) electrode interface, leading to a low- (high) resistance state under a positive (negative) bias. For the top electrode interface of Au/BTO, both the positive and negative ferroelectric bound charges can be perfectly screened by electrons and holes, respectively, under both the positive and negative bias. Thus, the screening speeds can always be as fast as hundreds of picoseconds [19]; both SET and RESET speeds should be at the same time scale, so the top electrode interface of Au/BTO cannot account for the asymmetric resistive switching speed. However, for the bottom electrode interface of BTO/NSTO, the positive and ferroelectric bound charges can be screened by electrons and oxygen vacancies, respectively, under positive and negative bias. Actually, the oxygen vacancies can migrate across the BTO/NSTO interface, from BTO to NSTO (from NSTO to BTO) for polarization points towards (away from) NSTO under a positive (negative) bias applied on the top electrode. When the ferroelectric polarization is pointed from top to bottom electrode, electrons are needed to screen the positive ferroelectric bound charges at the bottom electrode interface; thus, only the movement speed of electrons will affect the SET speed in the resistive switching process. When the ferroelectric polarization is pointed from bottom to top electrode, oxygen vacancies are needed to screen the negative ferroelectric bound charges at the bottom electrode interface; thus, the movement speed of oxygen vacancies will restrict the RESET speed in the resistive switching process. Since the migration of oxygen vacancies takes much longer time than that of electrons, the SET speed restricted by electrons will be much faster than the RESET speed restricted by oxygen vacancies, which is consistent with our observation. Furthermore, the transition between electronic screening and oxygen vacancy screening has also been observed in the BiFeO_3_/La_0.7_Sr_0.3_MnO_3_ interface [20], which further confirms the proposed mechanism for the asymmetric resistive switching in the present work.

## Conclusions

In conclusion, asymmetric resistive switching time is observed in BTO/NSTO heterojunctions. The pulse duration required for RESET operation is four orders longer than that for the SET process. The positive and negative ferroelectric bound charges screened by electrons and oxygen vacancies at the BTO/NSTO interface play an important role at a positive and negative bias, respectively. The process of electron screening is much faster than that of oxygen vacancies, so the SET transition (HRS to LRS) induced by positive bias is much faster than the RESET transition (LRS to HRS) induced by negative bias. Furthermore, this switch exhibits fast SET and slow RESET transition, which may have potential applications in some special regions.

## Abbreviations

BTO: BaTiO_3_
HRS: High-resistance state
LRS: Low-resistance state
NSTO: Nb:SrTiO_3_
PFM: Piezoresponse force microscopy
SKPM: Scanning Kelvin probe microscopy
